# Exocrine pancreatic function is preserved in systemic sclerosis

**DOI:** 10.1186/s13075-019-1840-z

**Published:** 2019-02-12

**Authors:** Gracijela Bozovic, Rille Pullerits, Arne Ståhl, Kristina Ydström, Daniel Wenger, Jan Marsal, Pontus Thulin, Kristofer Andréasson

**Affiliations:** 1 Department of Medical Imaging and Physiology, Skåne University Hospital, Lund, Sweden; 2 Department of Rheumatology and Inflammation Research, Sahlgrenska Academy at University of Gothenburg, Gothenburg, Sweden; 3 Department of Clinical Immunology and Transfusion Medicine, Sahlgrenska University Hospital, Gothenburg, Sweden; 4 Department of Medical Radiation Physics, Skåne University Hospital, Lund, Sweden; 5 Section of Orthopedics, Department of Clinical Sciences, Lund University, Lund, Sweden; 6 Section of Gastroenterology, Department of Clinical Sciences, Lund University, Lund, Sweden; 7 Section of Rheumatology, Department of Clinical Sciences, Lund University, 221 85 Lund, Sweden

**Keywords:** Pancreas, Systemic sclerosis, Fecal elastase, Malnutrition

## Abstract

**Background:**

Systemic sclerosis (SSc) has been suggested to cause exocrine pancreatic dysfunction. However, a case-control-based autopsy study failed to associate systemic sclerosis with any pancreatic histopathology. The primary objective of this study was to examine the exocrine pancreatic function in consecutive SSc patients in relation to an age- and sex-matched control group. A secondary objective was to relate exocrine pancreatic function to radiological, laboratory, and clinical SSc characteristics.

**Methods:**

One hundred twelve consecutive patients fulfilling the 2013 American Congress of Rheumatology/European League Against Rheumatism criteria for SSc and 52 control subjects were matched for sex and age. Exocrine pancreatic function was assessed by ELISA-based measurement of fecal elastase, and levels ≤ 200 μg/g were considered pathological, i.e., representing exocrine pancreatic insufficiency. Patients were characterized regarding SSc manifestations including gastrointestinal and hepatobiliary function, by use of laboratory and clinical examinations. Pancreas parenchyma characteristics were evaluated by high-resolution computer tomography (HRCT).

**Results:**

A similar proportion of subjects exhibited pathological levels of fecal elastase among SSc patients (6/112; 5.4%) and control subjects (3/52; 5.8%). Patients with fecal elastase ≤ 200 μg/g did not differ from other SSc patients with respect to laboratory and clinical characteristics, including malnutrition. SSc subjects with low levels of fecal elastase displayed significantly lower pancreas attenuation on HRCT examinations compared to the control subjects.

**Conclusions:**

In this study encompassing 112 consecutive SSc patients and 52 matched control subjects, we were unable to associate systemic sclerosis with clinically significant exocrine pancreatic dysfunction.

**Electronic supplementary material:**

The online version of this article (10.1186/s13075-019-1840-z) contains supplementary material, which is available to authorized users.

## Introduction

Systemic sclerosis (SSc) is a heterogeneous systemic disease characterized by the development of autoimmunity, vasculopathy, and multiorganic fibrosis. Involvement of the gastrointestinal (GI) tract is common, affecting up to 90% of patients, and is a significant contributor to both morbidity and mortality [1]. Malnutrition is common, and its etiology is multifactorial and incompletely understood. Factors that may play a role include reduced appetite, poor functional status of the hands and fingers, esophageal and GI dysmotility, and small intestinal bacterial overgrowth [2]. SSc has been linked to exocrine pancreatic insufficiency (EPI), and EPI has been suggested to contribute to malnutrition in SSc [1, 3–6].

EPI is easily and efficiently treated with pancreatic enzyme replacement therapy [7]. Consequently, it is important to identify SSc patients suffering from this disorder. During the last decades, the measurement of fecal elastase (FE) has been established as a reliable method to screen for clinically significant EPI with a reported sensitivity above 90% [8]. Assessment of exocrine pancreatic function by FE measurement has also been recommended in the evaluation of SSc-associated malnutrition and steatorrhea [9, 10].

SSc-related pancreatic tissue pathologies are characterized by both inflammation and the conversion of functional parenchyma to a fibrous stroma, sometimes with the replacement of parenchyma with fat. Pancreatic fat can be quantified by non-contrast-enhanced computed tomography (CT), resulting in lower attenuation measured in Hounsfield units (HU) [11].

The purpose of this study was to investigate the prevalence of EPI in a consecutively assembled cohort of SSc patients in relation to an age- and sex-matched control group. As a secondary aim, we wanted to investigate if EPI in SSc was associated with any specific SSc characteristic or radiological alterations of the pancreas parenchyma.

## Methods

### Study population

Consecutive SSc patients at scheduled routine visits to our clinic were invited to participate in this study from April 2014 to June 2015. Age- and sex-matched control subjects were recruited from the staff of our clinics (*n* = 40), from the spouses of patients (*n* = 4), and from a neighboring orthopedic ward (*n* = 8). Patients and controls with concomitant pancreatic disease (including chronic pancreatitis and pancreatic cysts), a history of pancreatic surgery, or alcohol abuse were excluded from the study in order not to include patients with non-SSc-related EPI. Among the controls, subjects with rheumatic disease were also excluded. A separate control group was used for the radiological study, see below.

### Clinical characteristics

SSc was defined according to the 2013 American Congress of Rheumatology/European League Against Rheumatism (ACR/EULAR) criteria, and patients were subdivided into diffuse cutaneous SSc (dcSSc) and limited cutaneous SSc (lcSSc) [12, 13]. SSc disease duration was defined as years since the first non-Raynaud symptom of disease. Body mass index was recorded, and malnutrition was assessed according to the validated Malnutrition Universal Screening Tool (MUST) [14]. All patients were systematically questioned regarding the following GI symptoms: heartburn (dyspepsia), dysphagia, diarrhea, and/or constipation. These were recorded as present or not. Lung fibrosis was defined as the presence of fibrosis on high-resolution computed tomography (HRCT) as specified in the ACR/EULAR criteria [12]. Esophageal function was assessed by barium cineradiography and graded 0 (normal) to 2 (aperistalsis) since this investigation has been suggested to be a marker of GI manifestations of SSc [15].

### Laboratory analyses

FE was measured with ELISA utilizing a monoclonal antibody towards pancreatic elastase 1 (Schebo Biotech, Giessen, Germany). Samples from control subjects and patients were analyzed in duplicates on the same ELISA plates. According to the manufacturer and the literature [7, 8], levels below 15 μg/g were considered indicative of severe pancreatic dysfunction while levels between 15 and 200 μg/g were categorized as possible insufficiency. With a suggested cutoff of 200 μg/g, this analysis has been shown to identify EPI with high sensitivity and specificity [8]. F-calprotectin, a marker of GI inflammation that has previously been associated with GI manifestations of SSc, was measured with ELISA (Calpro, Lysaker, Norway) [16]. Markers of hepatobiliary function (aspartate aminotransferase, alanin aminotransferase, alkaline phosphatase, gamma-glutamyltransferase) were assessed as well as pancreatic amylase. Prealbumin (transthyretin) and albumin were measured as markers of malnutrition. Prealbumin levels below 200 mg/l were considered indicative of malnutrition [17]. Vitamin D was measured since low levels of this vitamin have been associated with both SSc and EPI [18, 19].

### Radiological assessment of the pancreas

All patients had been examined with a mandatory HRCT in search for pulmonary involvement of SSc. Although focused on the lungs, it includes all or most of the pancreas since the basal parts of the lungs extend over the upper abdomen. We included a separate control group for the radiology analysis. These control subjects were randomly selected from the radiologist’s workflow during a 6-month period and matched for sex and age ± 6 years apart, with the exception of two patients. They had been referred to the radiologist for HRCT and did not have any concomitant pancreatic disease, diabetes mellitus, a history of pancreatic surgery, or alcohol abuse. Radiology subjects were divided into three groups, each pre-specified to encompass at least seven subjects:

1. SSc patients with low FE levels (*n* = 7; FE ≤ 210 μg/g);
2. Age- and sex-matched SSc patients with normal levels of FE (*n* = 21);
3. Age- and sex-matched control subjects without SSc (*n* = 21).

The HRCT examinations were performed with routine scan settings and reconstruction parameters on CT scanners. HRCT parameters are summarized in Additional file 1: Table S1. All scans were done with 120 kV using dose modulation to optimize the image quality according to patient size. Reconstructions were done with 3 mm slice thickness and with kernel/filter according to Additional file 1: Table S1.

**Fig. 1:**
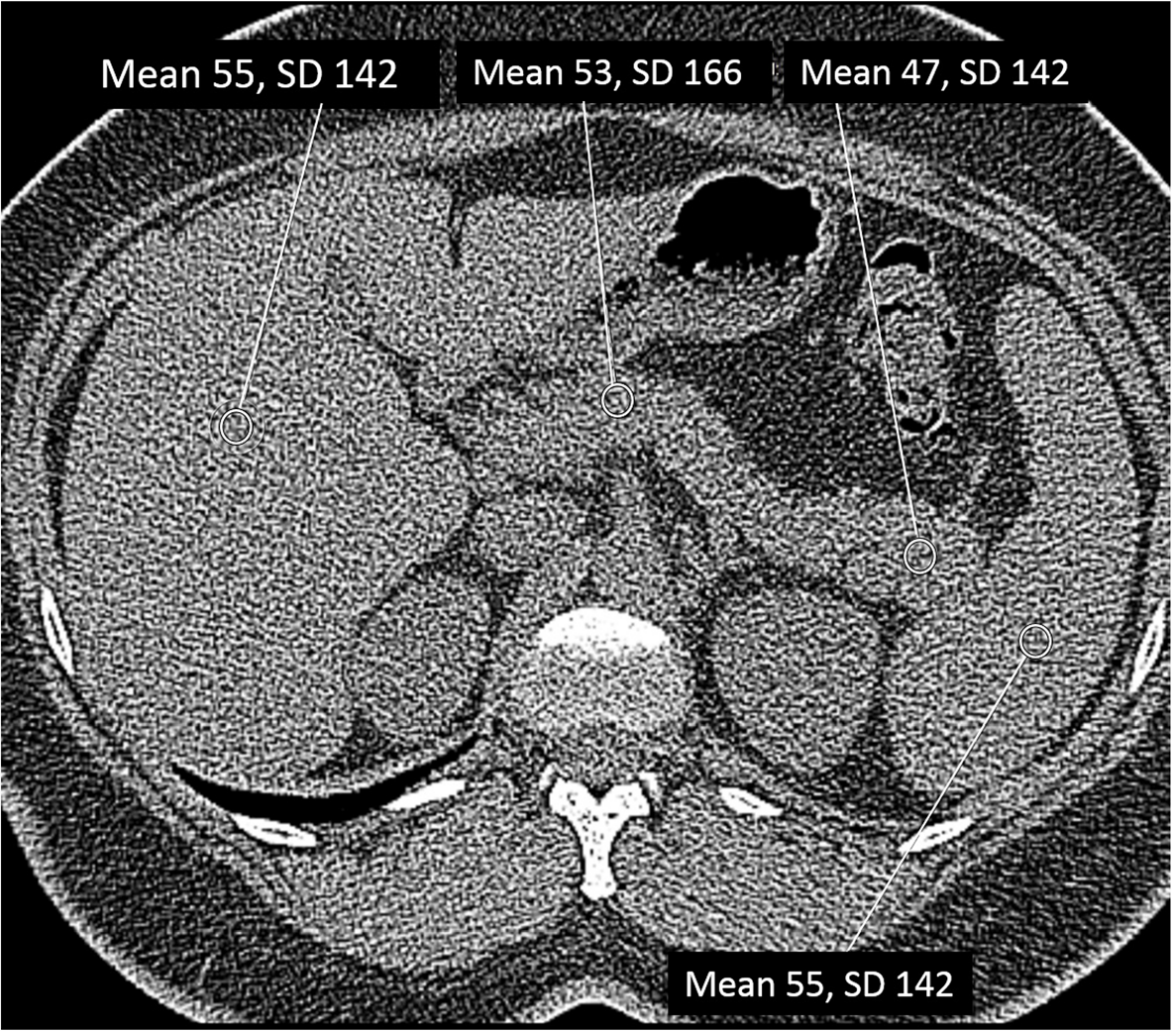
HRCT of the chest including the upper abdomen. The image shows from left to right a region of interest for measurement of the CT number (Hounsfield units) placed in the liver, pancreatic body and tail, and spleen respectively. The measurements of the spleen were used for normalization of attenuation (Hounsfield units) to compensate for variability between different CT machines

Retrospectively, the CT number (HU value) was measured by one radiologist blinded for the clinical data. A circular region of interest (ROI) up to 1 cm in diameter was drawn in the pancreatic body and tail respectively, avoiding vascular structures and cysts if present (Fig. 1). Furthermore, a similar ROI was placed in the spleen as a reference for normalization of attenuation to compensate for the variability between different CT machines [20].

### Statistics

Non-parametric statistics and Fischer’s exact test were consistently used in this study when describing and comparing FE in the patient and control group, as well as when analyzing FE in relation to patient data. Spearman’s rank-order correlation was used when relating FE to F-calprotectin and esophageal function. Parametric statistics were used when analyzing the radiological examinations. *p* < 0.05 was considered significant.

### Ethics

The study was approved by the Regional Ethics Review Board, Lund, Sweden, reference number 2011/596. Informed written consent was obtained from all subjects before study inclusion, and the study conformed to the ethical guidelines of the Declaration of Helsinki.

## Results

### F-elastase

**Fig. 2:**
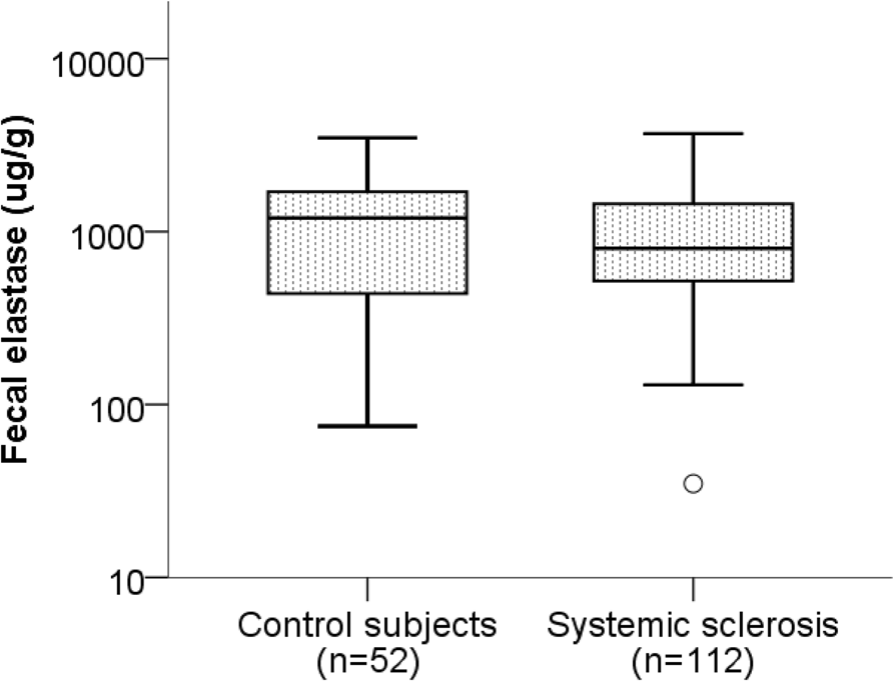
Fecal elastase levels in systemic sclerosis and control subjects. Box plot indicating fecal elastase levels in patients with systemic sclerosis and age- and sex-matched controls

The characteristics of patients and control subjects are described in Table 1. A minority of the SSc patients (6/112; 5.4%) exhibited FE levels ≤ 200 μg/g, of which no one presented levels indicative of severe dysfunction (< 15 μg/g). In the control group, 3/52 (5.8%) had FE levels ≤ 200 μg/g, of which no one had levels below 15 μg/g, which was not statistically different from patients with SSc (*p* = 1.00). Median (interquartile range [IQR]) levels of FE were similar between patients (800 [515–1475] μg/g) and control subjects (1200 [435–1700] μg/g; *p* = 0.189), as shown in Fig. 2.

**Table 1:** Patient and control subjects characteristics

	SSc subjects (*n* = 112)	Control subjects (*n* = 52)
Age (years)	62 (50, 69)	62 (51, 66)
Sex (female/male)	89/23 (3.9:1)	41/11 (3.7:1)
Disease duration (years)	7 (3, 15)	
Disease subtype (dcSSc/lcSSc)	26/86 (1:3.3)	
ANA positive (*n* %)	105 (94%)	
ACA positive (*n* %)	39 (35%)	
ATA positive (*n* %)	20 (18%)	
ARA positive (*n* %)	10 (9%)	
Lung fibrosis (*n* %)	37 (33%)	
Cineradiography (normal; mild to moderate pathology; aperistalsis) (*n* = 110)	22; 80; 8	
MUST score (0; 1; 2)	94; 15; 3	
Prealbumin < 200 mg/l (*n* %)*	16 (24%)	
Heartburn^†^	59 (53%)	
Dysphagia^†^	47 (42%)	
Diarrhea^†^	12 (11%)	
Constipation^†^	14 (13%)	

Values are expressed as median (interquartile range) if not otherwise stated

*dcSSc* diffuse cutaneous systemic sclerosis, *lcSSc* limited cutaneous systemic sclerosis, *ACA* anti-centromere antibodies, *ATA* anti-topoisomerase antibodies, *ARA* anti-RNA polymerase 3 antibodies, *MUST* Malnutrition Universal Screening Tool [13]

*Prealbumin analyzed in 68 patients

^†^Data available on 111 patients

### Clinical characteristics

Three of the six patients with low FE levels had dcSSc. This disease subtype was not statistically overrepresented compared to the lcSSc (*p* = 0.137).

None of the 13 patients with a body mass index < 20 and none of the 18 patients with a MUST score ≥ 1 (indicating malnutrition) had FE ≤ 200 μg/g. Median FE levels did not differ between patients with and without pathological MUST scores (940 [590–1850] and 800 [510–1400] μg/g, respectively; *p* = 0.428). Also, median FE levels did not differ between those with and without pathological prealbumin levels (675 [423–1065] and 780 [565–1400] μg/g, respectively; *p* = 0.284, *n* = 68). Only 1 of 16 patients with pathological prealbumin levels had FE ≤ 200 μg/g. The presence of GI symptoms was not associated with pathological FE testing (*p* = 0.377, *p* = 0.648, *p* = 0.562, and *p*= 0.691 for heartburn, dysphagia, diarrhea, and constipation, respectively). Anti-mitochondrial antibodies were present in 7 patients, 5 patients had a diagnosis of primary biliary cirrhosis, and 3 had diabetes mellitus; none of these had pathological FE testing.

FE levels were not associated with intestinal inflammation as assessed by F-calprotectin (*r*_s_ = 0.00, *p* = 0.952) or intestinal dysmotility as assessed by cineradiography (*r*_s_ = − 0.08, *p* = 0.422). FE ≤ 200 μg/g did not associate with laboratory markers of hepatobiliary function, pancreatic amylase, SSc disease duration, or antibody profile (Table 2).

**Table 2:** Laboratory and clinical characteristics of patients with and without pathological levels of fecal elastase

	ALT (U/L)	AST (U/L)	GGT (U/L)	ALP (U/L)	Pancreatic amylase (U/L)	Calcium (mmol/l)	Magnesium (mmol/l)	Albumin (g/l)	Prealbumin (g/l)	Vitamin D3 (nmol/l)	Disease duration (years)	Age (years)	ACA (*n*)	ATA (*n*)	ARA (*n*)
FE ≤ 200 μg/g (*n* = 6)	25 (16–41)	29 (22, 40)	55 (22, 156)	71 (71,881.2)	24 (22, 32)	2.4 (2.3, 2.5)	0.93 (0.77, 1.1)	40 (37, 42)	0.33 (0.19, 0.36)	48 (29, 65)	5 (1, 15)	70 (57, 76)	1	1	1
FE > 200 μg/g (*n* = 106)	19 (14, 24)	24 (21, 29)	25 (17, 0.46)	71 (52, 81)	25 (18, 0.38)	2.3 (2.3, 2.4)	0.82 (0.77, 0.86)	39 (36, 41)	0.25 (0.2, 0.3)	70 (45, 78)	7(3, 15)	60 (61, 69)	38	19	9

Systemic sclerosis patients with pathological FE testing did not differ compared to other patients with regard to laboratory markers of liver function and malnutrition, disease duration, age, and antibody profile. *p* > 0.05 for all variables when comparing patients with FE ≤ 200 μg/g to patients with FE > 200 μg/g. Values are given as median (interquartile range)

*FE* fecal elastase, *ALT* alanin aminotransferase, *AST* aspartate aminotransferase, *GGT* gamma-glutamyltransferase, *ALP* alkaline phosphatase, *ACA* anti-centromere antibodies, *ATA* anti-topoisomerase antibodies, *ARA* anti-RNA polymerase III antibodies

### Radiological assessment

**Fig. 3:**
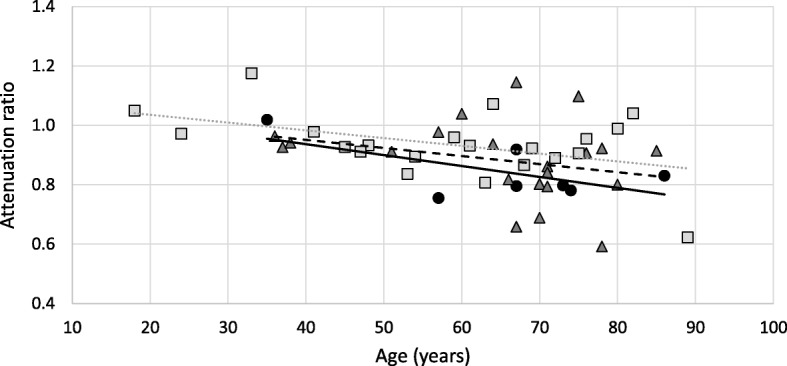
Pancreas attenuation in systemic sclerosis and control subjects. Graph showing the ratio of mean attenuation (CT number, Hounsfield units) for the pancreas (body + tail) in relation to the spleen in patients with and without fecal elastase values ≤ 210 μg/g and in control subjects, as a function of age. Group I (SSc with low levels of fecal elastase), circle/full black line; group II (age and sex-matched control subjects with SSc), triangle/dashed line; group III (age- and sex-matched control subjects without SSc): square/dotted line. The ratio shows an expected decrease with age. Significant difference was seen between group I and croup III (*p* = 0.024)

In this analysis, 28 patients and 21 control subjects were analyzed. Controls were patients who underwent a HRCT for chronic obstructive pulmonary disease, pulmonary fibrosis, or bronchiectasis. The HRCT examination was done within 1 year of the FE sampling in 22 of the 28 subjects with SSc. The median (IQR) age in the control group (*n* = 21) was 63 (47–72) years, similar to the median age of the SSc subjects with low FE (*n* = 7, median age 67 [62–74] years) and normal FE (*n* = 21, median age 71 [58–73] years) who were subject to radiological analysis (*p* = 0.406). We identified an age-dependent variation in pancreas attenuation both in the SSc subjects (*r* = − 0.39, *p* = 0.041) and the control subjects (*r* = − 0.45, *p* = 0.044). Pancreas attenuation, normalized in reference to the spleen, was significantly lower in SSc patients with low levels of FE compared to control subjects (0.798 vs. 0.932; *p* = 0.024), as shown in Fig. 3. However, SSc patients with normal levels of FE did not express significantly different attenuation compared to control subjects (0.910 vs. 0.932, *p* = 0.201).

## Discussion

Malnutrition afflicts approximately 18% of patients with SSc, is hard to manage, and is associated with both significant morbidity and mortality [2]. EPI is an important potential cause of malnutrition since it can be easily and efficiently treated with specific replacement therapy. In order to assess the prevalence of clinically significant EPI in SSc, we have examined 112 consecutive SSc patients by measurements of FE and related these findings to 52 age- and sex-matched controls. Low levels of FE were uncommon in both the SSc and the control groups. These data indicate that EPI is not a major cause of SSc-related malnutrition.

The gold standard for assessing EPI is the secretin-cerulein test. This test is invasive and cumbersome and not recommended for screening purposes [7]. FE is a pancreas-specific enzyme that is not degraded during intestinal transport. The enzyme is inert to degradation also when stored in room temperature. It can be successfully measured with ELISA and has proven to have limited intraindividual variability [8, 21]. FE measurement is superior to other indirect tests of pancreatic function including the fecal chymotrypsin test and the ^13^C-mixed triglyceride breath test. Measurement of FE with the monoclonal ELISA used in this study has a sensitivity and specificity above 90% in identifying EPI. Consequently, FE measurement has been established as an alternative to secretin-cerulein test when screening for EPI in both research and clinical settings [8, 22]. Even if FE has been suggested to be of value in the evaluation of SSc-related malnutrition [9], to our knowledge, FE has not previously been studied in SSc.

Conflicting data exists regarding the prevalence of EPI in SSc. A case-control autopsy study from 1969 failed to identify SSc-specific pancreas pathology in 58 SSc patients [23]. Smaller functional studies have suggested that EPI may be prevalent in SSc [3–6, 24], although none of them included a control group. The clinical relevance of the alterations identified in these studies is unclear since mild EPI is often asymptomatic [7]. Also, the generalizability of the larger of these studies (*n* = 31) is questionable since it comprised “a highly selective group of patients deliberately sequestered by virtue of the gastrointestinal complaints” [3].

We observed that 77% of our patients had lcSSc. Although not statistically significant, we noted that EPI was more common among patients with dcSSc. In the study by Shawis et. al, encompassing five dcSSc and six lcSSc subjects, two out of three patients with EPI had lcSSc [6]. Earlier works on EPI in SSc were published before these classification subtypes were established [13].

In our cohort, a pathological MUST score was present in 16% of the subjects and pathological prealbumin levels observed in 24% of the patients studied. These figures are similar to what has previously been reported in SSc and indicate that malnutrition was indeed prevalent in our cohort [14, 17]. Still, in this study, we were unable to associate malnutrition to EPI.

It was beyond the scope of this study to investigate other, non-pancreatic causes of malnutrition. Previous studies have suggested a complex mixture of SSc-related complications including GI dysmotility and small intestinal bacterial overgrowth, systemic inflammation, and extraintestinal manifestations to cause malnutrition in SSc [2]. Our results indicate that exocrine pancreatic dysfunction is not an important factor behind SSc-related malnutrition compared to the ones presented above.

In order to further investigate the pancreas in relation to SSc, patients with and without pathological FE were retrospectively studied using CT. The HRCT examinations identified an age-dependent decline in pancreatic attenuation in keeping with previous studies [11], but also a statistically significant difference in pancreatic attenuation in the subgroup of patients with low levels of FE compared to control subjects. The lower attenuation is likely caused by the replacement of exocrine pancreatic tissue with fat (pancreatic lipomatosis), possibly reflecting a destructive inflammatory process with increased parenchymal turnover [11]. Whether these data reflect an SSc-specific process remains to be elucidated. We were unable to find any similar differences when comparing SSc patients with normal FE levels to control subjects. HRCT is not the optimal imaging modality to assess the pancreas. Our patients were examined with a mandatory chest HRCT because of suspected lung fibrosis, an examination that usually includes all or most of the pancreas due to the anatomy of the lung. With respect to the radiation risks associated with CT examinations, we chose not to assess additional radiological examination but benefit from already existing ones. Further studies using magnetic resonance imaging and endoscopy, as well as autopsy studies, are warranted to understand if and how the pancreas may be affected in SSc.

## Conclusions

Our knowledge on any SSc-specific pathobiology of the pancreas is limited. In order to determine the prevalence of EPI in SSc, we have investigated a fairly large number of consecutive SSc patients and age- and sex-matched control subjects with a validated and sensitive marker of EPI and by HRCT. The radiological analyses might suggest that SSc in some cases may manifest itself in the pancreas but on the whole; our study indicates that exocrine pancreatic function is usually preserved in SSc.

## Additional file

Additional file 1: **Table S1.** High-resolution tomography parameters of machines used in this study. (DOCX 13 kb)

## Abbreviations

ACR/EULAR: American Congress of Rheumatology/European League Against Rheumatism
CT: Computer tomography
dcSSc: Diffuse cutaneous systemic sclerosis
EPI: Exocrine pancreas insufficiency
FE: Fecal elastase
GI: Gastrointestinal
HRCT: High-resolution computer tomography
HU: Hounsfield units
IQR: Interquartile range
lcSSc: Limited cutaneous systemic sclerosis
MUS: Malnutrition Universal Screening Tool
ROI: Region of interest
SSc: Systemic sclerosis
