# Supplementary material for: Exocrine pancreatic function is preserved in systemic sclerosis
Source: Arthritis Res Ther. 2019 Feb 12;21:52. doi: 10.1186/s13075-019-1840-z (PMC6373050; doi:10.1186/s13075-019-1840-z)
Supplement: Supplementary file 1 — Table S1. High-resolution tomography parameters of machines used in this study. (DOCX 13 kb) [file 13075_2019_1840_MOESM1_ESM.docx]

**Table S1** *High resolution tomography parameters of machines used in this study.*

| **Scanner** | **Recon kernel** |
| --- | --- |
| Siemens Somatom Definition Edge | i46f |
| Siemens Flash | b80f |
| Philips Ingenuity | L |
| Philips Brilliance 16 | D |
| Philips iCF | L, D, iDose 1 |
